# Supplementary material for: The Evolution and Characterization of the RNA Interference Pathways in Lophotrochozoa
Source: Genome Biol Evol. 2024 May 7;16(5):evae098. doi: 10.1093/gbe/evae098 (PMC11114477; doi:10.1093/gbe/evae098)
Supplement: evae098_Supplementary_Data [file evae098_supplementary_data.zip › Supplementary_materials.zip!/selected_miRNAs/NoGe_selected_miRNAs/mirdeep_pdf_files/NMRB01000795.1_Notospermus_geniculatus_strain_Ushimado_scaffold795,_whole_genome_shotgun_sequence_20953.pdf]

Provisional ID : NMRB01000795.1_Notospermus_geniculatus_strain_Ushimado_scaffold795,_whole_genome_shotgun_sequence_20953

Score total	:	179881.7
Score for star read(s)	:	3.9
Score for read counts	:	179876.6
Score for mfe	:	1.8
Score for randfold	:	0
Score for cons. seed	:	-0.6
Total read count	:	352832
Mature read count	:	352660
Loop read count	:	0
Star read count	:	172

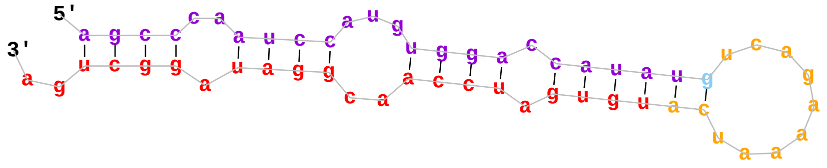

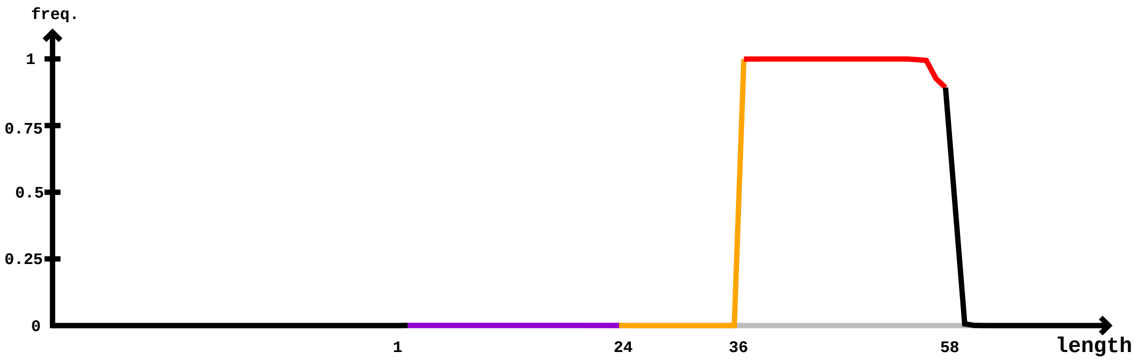


```
                                  Star                             Mature
5'- gaggccccuaucaugccucggccuguuuuccuuucagcccaauccauguggaccauaugucagaaaaucaugugauccaacggauaggcugaaagcaaaacaaaaugc   -3'   obs
    gaggccccuaucaugccucggccuguuuuccuuucagcccaauccauguggaccauaugucagaaaaucaugugauccaacggauaggcugaaagcaaaacaaaaugc         exp
    (((((.........)))))....((((((.(((((((((..((((...((((.((((((........)))))).)))).)))).))))))))).))))))......   reads    mm    sample
    ..................................aAcccaauccauguggac.......................................................   1        1     Mm3
    ..................................agcccaauccauguggacc......................................................   1        0     Mm3
    ..................................agcccaauccauguggaccau....................................................   3        0     Mm3
    ..................................agcccaauccauguggaccaua...................................................   4        0     Mm3
    ..................................agcccaauccauguggaccauau..................................................   13       0     Mm3
    ..................................agcccaauccauguggaAcauau..................................................   1        1     Mm3
    ..................................agcccaauccauguggaccauaug.................................................   1        0     Mm3
    ..................................agcccaauccauguggaccauauU.................................................   4        1     Mm3
    ...................................gcccaauccauguggaccau....................................................   1        0     Mm3
    ...................................gcccaauccauguggaccauau..................................................   1        0     Mm3
    ..............................................................aaaaucaugugauccaacggau.......................   2        0     Mm3
    ...................................................................caugugauccaacggauaggcu..................   1        0     Mm3
    ....................................................................Cugugauccaacggauaggcuga................   2        1     Mm3
    .....................................................................ugugauccaacggauagC....................   1        1     Mm3
    .....................................................................ugugauccaacggauagA....................   28       1     Mm3
    .....................................................................ugugauccaacggauagg....................   10       0     Mm3
    .....................................................................ugugauccaacggauagU....................   107      1     Mm3
    .....................................................................ugugauccaacggauaAgc...................   1        1     Mm3
    .....................................................................ugugauccaacggauagUc...................   1        1     Mm3
    .....................................................................ugugauccaacggauaggA...................   5        1     Mm3
    .....................................................................ugugauccaacggauaUgc...................   1        1     Mm3
    .....................................................................ugugauccaacggauaggU...................   33       1     Mm3
    .....................................................................ugugauccaacggauaggc...................   120      0     Mm3
    .....................................................................ugugauccaacggauGggc...................   1        1     Mm3
    .....................................................................Cgugauccaacggauaggcu..................   2        1     Mm3
    .....................................................................ugugauUcaacggauaggcu..................   1        1     Mm3
    .....................................................................ugugauccaacggauaggUu..................   2        1     Mm3
    .....................................................................ugugCuccaacggauaggcu..................   2        1     Mm3
    .....................................................................ugugauccaacggauaCgcu..................   9        1     Mm3
    .....................................................................ugugauccaacgAauaggcu..................   1        1     Mm3
    .....................................................................uAugauccaacggauaggcu..................   2        1     Mm3
    .....................................................................ugugauccaaAggauaggcu..................   9        1     Mm3
    .....................................................................ugugauccaacggauaggcu..................   5231     0     Mm3
```

```
                                   Star                             Mature
gaggccccuaucaugccucggccuguuuuccuuucagcccaauccauguggaccauaugucagaaaaucaugugauccaacggauaggcugaaagcaaaacaaaaugc
......................................................................ugugauccUacggauaggcu..................   1      1    Mm3
......................................................................ugugauNcaacggauaggcu..................   1      1    Mm3
......................................................................ugugauccaacggauaggcA..................   25     1    Mm3
......................................................................Agugauccaacggauaggcu..................   1      1    Mm3
......................................................................ugugauccaacgUauaggcu..................   1      1    Mm3
......................................................................ugugauAcaacggauaggcu..................   1      1    Mm3
......................................................................Ngugauccaacggauaggcu..................   3      1    Mm3
......................................................................ugGgauccaacggauaggcu..................   1      1    Mm3
......................................................................ugugauccaacggauGggcu..................   61     1    Mm3
......................................................................ugugauccaacggauUggcu..................   1      1    Mm3
......................................................................ugAgauccaacggauaggcu..................   2      1    Mm3
......................................................................ugugauccaGcggauaggcu..................   1      1    Mm3
......................................................................uguAauccaacggauaggcu..................   1      1    Mm3
......................................................................ugugauccaaUggauaggcu..................   8      1    Mm3
......................................................................ugugauccaaGggauaggcu..................   3      1    Mm3
......................................................................ugugauccaacggauaggcG..................   200    1    Mm3
......................................................................ugugauccaacggaCaggcu..................   1      1    Mm3
......................................................................ugugauccaacggauagCcu..................   30     1    Mm3
......................................................................ugugauccaacggauCggcu..................   1      1    Mm3
......................................................................ugugauccaacggauagUcu..................   22     1    Mm3
......................................................................ugugauccaacggaGaggcu..................   1      1    Mm3
......................................................................ugugaCccaacggauaggcu..................   1      1    Mm3
......................................................................ugugauccaCcggauaggcu..................   1      1    Mm3
......................................................................ugugauccaacggauaAgcu..................   10     1    Mm3
......................................................................ugugauccaacggaAaggcu..................   1      1    Mm3
......................................................................ugugauccaacggauagAcu..................   33     1    Mm3
......................................................................ugCgauccaacggauaggcu..................   2      1    Mm3
......................................................................ugugauccaacggauaggcC..................   18     1    Mm3
......................................................................ugugauccaacggGuaggcu..................   1      1    Mm3
......................................................................ugugauccaacggauaUgcu..................   7      1    Mm3
......................................................................ugugauccaacggauaCgcug.................   3      1    Mm3
......................................................................ugugauccaacggauagAcug.................   7      1    Mm3
......................................................................ugugauccaacggauaggcug.................   1346   0    Mm3
......................................................................ugugauccaaAggauaggcug.................   3      1    Mm3
......................................................................ugugauccaacggauaAgcug.................   4      1    Mm3
......................................................................ugugaNccaacggauaggcug.................   1      1    Mm3
......................................................................ugugauccaacggauaggcGg.................   21     1    Mm3
......................................................................Agugauccaacggauaggcug.................   1      1    Mm3
......................................................................ugugauccaacggauaUgcug.................   2      1    Mm3
......................................................................ugugauccaacggauaggcuA.................   75     1    Mm3
......................................................................ugGgauccaacggauaggcug.................   2      1    Mm3
......................................................................ugugauccaacggauaggcuU.................   66     1    Mm3
......................................................................ugugauccaacggauagCcug.................   4      1    Mm3
......................................................................ugugauccaacggauGggcug.................   24     1    Mm3
......................................................................ugugauccaacggauagUcug.................   10     1    Mm3
......................................................................ugugauccaaGggauaggcug.................   3      1    Mm3
......................................................................uCugauccaacggauaggcug.................   1      1    Mm3
......................................................................ugugGuccaacggauaggcug.................   1      1    Mm3
......................................................................ugugauccaacggaCaggcug.................   1      1    Mm3
......................................................................ugugauccaaUggauaggcug.................   1      1    Mm3
......................................................................ugAgauccaacggauaggcuga................   2      1    Mm3
......................................................................ugugauccUacggauaggcuga................   6      1    Mm3
......................................................................ugugauccaacggauUggcuga................   4      1    Mm3
......................................................................ugugauccaacCgauaggcuga................   1      1    Mm3
......................................................................ugugGuccaacggauaggcuga................   3      1    Mm3
......................................................................ugugauccaacggauaggcCga................   2      1    Mm3
......................................................................ugugauccaaGggauaggcuga................   19     1    Mm3
......................................................................ugugUuccaacggauaggcuga................   2      1    Mm3
......................................................................ugugauccaacggauaggcugC................   23     1    Mm3
......................................................................ugugaAccaacggauaggcuga................   4      1    Mm3
......................................................................ugugauUcaacggauaggcuga................   1      1    Mm3
......................................................................ugugauccaGcggauaggcuga................   3      1    Mm3
......................................................................uguAauccaacggauaggcuga................   2      1    Mm3
......................................................................ugugauccaaUggauaggcuga................   18     1    Mm3
......................................................................ugugauccaacgCauaggcuga................   2      1    Mm3
......................................................................ugugauccaacggauaggAuga................   1      1    Mm3
......................................................................ugugauccaacggauaggcugU................   6758   1    Mm3
......................................................................uAugauccaacggauaggcuga................   11     1    Mm3
......................................................................uUugauccaacggauaggcuga................   1      1    Mm3
......................................................................ugugauccaacggauaUgcuga................   34     1    Mm3
```

Star **Mature**

```
gaggccccuaucaugccucggccuguuuuccuuucagcccaauccauguggaccauaugucagaaaaucaugugauccaacggauaggcugaaagcaaaacaaaaugc
......................................................................ugugauccaacggauagUcuga................    89      1    Mm3
......................................................................ugugauccaacggUuaggcuga................    4       1    Mm3
......................................................................ugugaucAaacggauaggcuga................    1       1    Mm3
......................................................................ugugauccaacggauaggUuga................    4       1    Mm3
......................................................................ugugauccaacggauGggcuga................    233     1    Mm3
......................................................................ugugauccaacggaAaggcuga................    1       1    Mm3
......................................................................ugugaCccaacggauaggcuga................    1       1    Mm3
......................................................................ugugauccGacggauaggcuga................    2       1    Mm3
......................................................................ugCgauccaacggauaggcuga................    4       1    Mm3
......................................................................ugugauccaCcggauaggcuga................    1       1    Mm3
......................................................................ugugauAcaacggauaggcuga................    2       1    Mm3
......................................................................ugugCuccaacggauaggcuga................    2       1    Mm3
......................................................................Agugauccaacggauaggcuga................    3       1    Mm3
......................................................................ugugauccaacggauaggcuga................    17641   0    Mm3
......................................................................ugugauccaacggauaggcuCa................    2       1    Mm3
......................................................................ugugauccaacggCuaggcuga................    3       1    Mm3
......................................................................ugugauccCacggauaggcuga................    1       1    Mm3
......................................................................ugGgauccaacggauaggcuga................    8       1    Mm3
......................................................................ugugauccaacAgauaggcuga................    4       1    Mm3
......................................................................ugugaucUaacggauaggcuga................    11      1    Mm3
......................................................................ugugauccaacgUauaggcuga................    1       1    Mm3
......................................................................ugugauccaacggauaggcugG................    259     1    Mm3
......................................................................Ngugauccaacggauaggcuga................    10      1    Mm3
......................................................................ugugauccaacggauaCgcuga................    46      1    Mm3
......................................................................ugugauccaacggaCaggcuga................    1       1    Mm3
......................................................................uguCauccaacggauaggcuga................    1       1    Mm3
......................................................................ugugauccaaAggauaggcuga................    23      1    Mm3
......................................................................ugugauccaUcggauaggcuga................    1       1    Mm3
......................................................................uguUauccaacggauaggcuga................    3       1    Mm3
......................................................................ugugauccaacggauaggcuAa................    15      1    Mm3
......................................................................ugugauccaacggauagAcuga................    105     1    Mm3
......................................................................uCugauccaacggauaggcuga................    3       1    Mm3
......................................................................ugugaucGaacggauaggcuga................    1       1    Mm3
......................................................................ugugauccaacggauaggcuUa................    3       1    Mm3
......................................................................ugugauccaacggauagCcuga................    95      1    Mm3
......................................................................ugugauccaacUgauaggcuga................    2       1    Mm3
......................................................................ugugaNccaacggauaggcuga................    2       1    Mm3
......................................................................ugugauccaacggauaAgcuga................    27      1    Mm3
......................................................................Ggugauccaacggauaggcuga................    1       1    Mm3
......................................................................ugugauccaacggauaggcugaG...............    6       1    Mm3
......................................................................ugugauccaacggauGggcugaa...............    7       1    Mm3
......................................................................ugugauccaacggauaggcuAaa...............    6       1    Mm3
......................................................................ugugauccaacggauaggcugCa...............    1       1    Mm3
......................................................................ugugauccaacggauaCgcugaa...............    6       1    Mm3
......................................................................ugugauccaacggauaAgcugaa...............    3       1    Mm3
......................................................................ugugauccaaGggauaggcugaa...............    3       1    Mm3
......................................................................ugugaucGaacggauaggcugaa...............    1       1    Mm3
......................................................................ugugauccaacggauaggUugaa...............    2       1    Mm3
......................................................................ugugauccaGcggauaggcugaa...............    1       1    Mm3
......................................................................ugugauccaacggauagAcugaa...............    4       1    Mm3
......................................................................ugugauccaacAgauaggcugaa...............    1       1    Mm3
......................................................................ugugauccaacggauagUcugaa...............    4       1    Mm3
......................................................................ugugauccaacggauaggcugaU...............    480     1    Mm3
......................................................................ugugaucUaacggauaggcugaa...............    1       1    Mm3
......................................................................ugugauccaacggauaggcugUa...............    67      1    Mm3
......................................................................ugugauccaaUggauaggcugaa...............    1       1    Mm3
......................................................................ugugauccaacggauaggcugaa...............    1281    0    Mm3
......................................................................uAugauccaacggauaggcugaa...............    1       1    Mm3
......................................................................ugugauccaacggauagCcugaa...............    3       1    Mm3
......................................................................ugugaNccaacggauaggcugaa...............    1       1    Mm3
......................................................................ugugauccaaAggauaggcugaa...............    2       1    Mm3
......................................................................ugugauccaacggauGggcugaaa..............    3       1    Mm3
......................................................................ugugauccaacggauaggcuAaaa..............    1       1    Mm3
......................................................................ugugauccaacggauaggcugaaG..............    6       1    Mm3
......................................................................ugugauccaacggauaggcugaaU..............    82      1    Mm3
......................................................................ugugauccaacggauaggcugaaC..............    1       1    Mm3
......................................................................ugugauUcaacggauaggcugaaa..............    1       1    Mm3
......................................................................ugugauccaacggauaggcugaUa..............    8       1    Mm3
......................................................................ugugauccaacggauaggcugUaa..............    10      1    Mm3
......................................................................ugugauccaacggauaggcugaaa..............    90      0    Mm3
```

Star **Mature**

```
gaggccccuaucaugccucggccuguuuuccuuucagcccaauccauguggaccauaugucagaaaaucaugugauccaacggauaggcugaaagcaaaacaaaaugc
......................................................................ugugauccaacggauaggcugaGa..............   3    1    Mm3
......................................................................ugugauccaacggauaAgcugaaa..............   1    1    Mm3
......................................................................ugugauccaacggauaggcugaaaU.............   15   1    Mm3
......................................................................ugugauccaacggauaggcugaaag.............   1    0    Mm3
......................................................................ugugauccaacggauaggcugaaaA.............   8    1    Mm3
......................................................................ugugauccaacggauaggcugaaagU............   1    1    Mm3
......................................................................ugugauccaacggauaggcugaaagAaaa.........   1    1    Mm3
.......................................................................gugauccaacggauaggcu..................   2    0    Mm3
.......................................................................gugauccaacUgauaggcug.................   1    1    Mm3
.......................................................................gugauccaacggauaggcug.................   2    0    Mm3
.......................................................................Uugauccaacggauaggcuga................   1    1    Mm3
.......................................................................gugauccaacggauGggcuga................   1    1    Mm3
.......................................................................gugauccaacggauaggcuga................   5    0    Mm3
........................................................................ugauccaacggauaggcug.................   1    0    Mm3
........................................................................ugauccaacggauaggcuga................   2    0    Mm3
........................................................................ugauccaacggauaggcugU................   1    1    Mm3
.........................................................................gauccaacggauaggcug.................   1    0    Mm3
.........................................................................gauccaacggauaggcuA.................   1    1    Mm3
..........................................................................auccaacggaAaggcuga................   1    1    Mm3
..........................................................................auccaacggauaggcuga................   1    0    Mm3

......................................................................ugugauccaacggauagU....................   1    1    Fm3
......................................................................ugugauccaacggauagA....................   3    1    Fm3
......................................................................ugugauccaacggauGggc...................   1    1    Fm3
......................................................................ugugauccaacggauaggU...................   2    1    Fm3
......................................................................ugugauccaacggauaggcu..................   17   0    Fm3
......................................................................ugugauccaacggauGggcu..................   4    1    Fm3
......................................................................ugugauccaacggauaggcug.................   7    0    Fm3
......................................................................ugugauccaacggauaggcuU.................   2    1    Fm3
......................................................................ugugauccaacggauaggAug.................   1    1    Fm3
......................................................................Ngugauccaacggauaggcuga................   1    1    Fm3
......................................................................ugugauccaacggauaUgcuga................   1    1    Fm3
......................................................................ugugauccaacggauGggcuga................   16   1    Fm3
......................................................................ugugauUcaacggauaggcuga................   1    1    Fm3
......................................................................ugugauccaacggauaCgcuga................   1    1    Fm3
......................................................................ugugauccaacggauaggcugU................   205  1    Fm3
......................................................................ugugauccaacggauagUcuga................   1    1    Fm3
......................................................................ugugauccaacggauaggAuga................   1    1    Fm3
......................................................................Ggugauccaacggauaggcuga................   1    1    Fm3
......................................................................ugugauccaacggauagCcuga................   3    1    Fm3
......................................................................ugCgauccaacggauaggcuga................   1    1    Fm3
......................................................................ugugauccaacggauaggcuga................   370  0    Fm3
......................................................................ugugauccaaUggauaggcuga................   1    1    Fm3
......................................................................ugugauccaacggauaggcugaa...............   16   0    Fm3
......................................................................ugugauccaacggauagCcugaa...............   1    1    Fm3
......................................................................ugugauccaaUggauaggcugaa...............   1    1    Fm3
......................................................................ugugauccaacggauaggcugaU...............   7    1    Fm3

...............................uuucagcccaauccauguggacc......................................................   1    0    Mm2
...................................agcccaauccauguggac.......................................................   1    0    Mm2
...................................agcccaauccauguggaccau....................................................   3    0    Mm2
...................................agcccaauccauguggaccauU...................................................   2    1    Mm2
...................................agcccaauccauguggaccaua...................................................   26   0    Mm2
...................................agcccaauccauguggaccauaA..................................................   1    1    Mm2
...................................agcccaauccauguggaccauau..................................................   53   0    Mm2
...................................agcccaauccaCguggaccauau..................................................   1    1    Mm2
...................................agcccaauccauguggaccauauU.................................................   4    1    Mm2
...................................agcccaauccauguggaccauaug.................................................   16   0    Mm2
...................................agcccaauccauguggaccauaugucaga............................................   4    0    Mm2
.....................................cccaauccauguggaccauaug.................................................   2    0    Mm2
...........................................................ucagaaaaucaugugauAcaacggauagg....................   1    1    Mm2
...........................................................ucagaaaaucaugugauccaacggauagg....................   1    0    Mm2
.................................................................aaucaugugauccaacggauaggU...................   3    1    Mm2
.................................................................aaucaugugauccaacggauaggcu..................   1    0    Mm2
.................................................................aaucaugugauccaacggauaggcuga................   3    0    Mm2
...................................................................ucaugugauccaacggauaggcuga................   1    0    Mm2
....................................................................caugugauccaacggauaggcu..................   6    0    Mm2
.....................................................................augugauccaacggauGggcu..................   1    1    Mm2
.....................................................................Cugugauccaacggauaggcu..................   1    1    Mm2
.....................................................................augugauccaacggauaggcugU................   1    1    Mm2
```

Star Mature

```
gaggccccuaucaugccucggccuguuuuccuuucagcccaauccauguggaccauaugucagaaaaucaugugauccaacggauaggcugaaagcaaaacaaaaugc
.....................................................................augugauccaacggauaggcuga................	7	0	Mm2
.....................................................................Cugugauccaacggauaggcuga................	7	1	Mm2
......................................................................ugugauccaacggauaUg....................	1	1	Mm2
......................................................................ugugauccaacggauagA....................	35	1	Mm2
......................................................................ugugauccaacggauagU....................	270	1	Mm2
......................................................................ugugauccaacggauGgg....................	4	1	Mm2
......................................................................ugugauccaacggauagg....................	34	0	Mm2
......................................................................ugugauccaacggauagUc...................	6	1	Mm2
......................................................................ugugauccaacggauagAc...................	2	1	Mm2
......................................................................ugugauccaacggaCaggc...................	1	1	Mm2
......................................................................ugugauccaacggauagCc...................	1	1	Mm2
......................................................................ugugauccaacggauaggA...................	9	1	Mm2
......................................................................ugugauccaacggauaggU...................	55	1	Mm2
......................................................................ugugauccaacggauaAgc...................	1	1	Mm2
......................................................................ugugauccaacggauaggc...................	440	0	Mm2
......................................................................ugugauccaacggauGggc...................	17	1	Mm2
......................................................................ugugauccaacggauaCgc...................	1	1	Mm2
......................................................................ugugauccaacggauaUgc...................	1	1	Mm2
......................................................................ugCgauccaacggauaggc...................	1	1	Mm2
......................................................................ugugauccaacggauaggcG..................	641	1	Mm2
......................................................................Agugauccaacggauaggcu..................	1	1	Mm2
......................................................................ugugaucUaacggauaggcu..................	2	1	Mm2
......................................................................ugugauAcaacggauaggcu..................	1	1	Mm2
......................................................................Cgugauccaacggauaggcu..................	2	1	Mm2
......................................................................ugugauccaacggauGggcu..................	449	1	Mm2
......................................................................ugugauccaacggauaAgcu..................	27	1	Mm2
......................................................................ugugauccaacgAauaggcu..................	1	1	Mm2
......................................................................ugugauccaacggCuaggcu..................	1	1	Mm2
......................................................................ugugauccaacAgauaggcu..................	3	1	Mm2
......................................................................uAugauccaacggauaggcu..................	6	1	Mm2
......................................................................Ngugauccaacggauaggcu..................	9	1	Mm2
......................................................................ugugauccaacggUuaggcu..................	1	1	Mm2
......................................................................ugugauccaacggauaCgcu..................	27	1	Mm2
......................................................................ugugauccaCcggauaggcu..................	1	1	Mm2
......................................................................ugugauccaacggaCaggcu..................	1	1	Mm2
......................................................................ugugauccaacggauaggcA..................	32	1	Mm2
......................................................................ugugauccaacggauagAcu..................	82	1	Mm2
......................................................................ugugauccaacggaGaggcu..................	1	1	Mm2
......................................................................uUugauccaacggauaggcu..................	2	1	Mm2
......................................................................ugGgauccaacggauaggcu..................	1	1	Mm2
......................................................................ugugauccaacggauaUgcu..................	17	1	Mm2
......................................................................ugugCuccaacggauaggcu..................	1	1	Mm2
......................................................................ugCgauccaacggauaggcu..................	2	1	Mm2
......................................................................uguAauccaacggauaggcu..................	2	1	Mm2
......................................................................ugAgauccaacggauaggcu..................	1	1	Mm2
......................................................................ugugauccaacggauaggcu..................	12137	0	Mm2
......................................................................uguUauccaacggauaggcu..................	2	1	Mm2
......................................................................ugugauccaaUggauaggcu..................	15	1	Mm2
......................................................................ugugauccaacgUauaggcu..................	1	1	Mm2
......................................................................ugugauccaacggauaggcC..................	16	1	Mm2
......................................................................ugugauccCacggauaggcu..................	2	1	Mm2
......................................................................ugugauccaacggauagUcu..................	48	1	Mm2
......................................................................ugugaAccaacggauaggcu..................	2	1	Mm2
......................................................................ugugauccaaGggauaggcu..................	18	1	Mm2
......................................................................ugugauccaacggauagCcu..................	59	1	Mm2
......................................................................ugugauccaacggauUggcu..................	2	1	Mm2
......................................................................uCugauccaacggauaggcu..................	2	1	Mm2
......................................................................ugugauccaacggaAaggcu..................	2	1	Mm2
......................................................................ugugaCccaacggauaggcu..................	2	1	Mm2
......................................................................ugugaNccaacggauaggcu..................	2	1	Mm2
......................................................................ugugauGcaacggauaggcu..................	1	1	Mm2
......................................................................ugugauccGacggauaggcu..................	2	1	Mm2
......................................................................ugugaucGaacggauaggcu..................	1	1	Mm2
......................................................................ugugauccUacggauaggcu..................	10	1	Mm2
......................................................................ugugauccaaAggauaggcu..................	18	1	Mm2
......................................................................uguCauccaacggauaggcu..................	1	1	Mm2
......................................................................ugugauccaacggauagCcug.................	24	1	Mm2
......................................................................ugGgauccaacggauaggcug.................	2	1	Mm2
......................................................................ugugauccaacggauagUcug.................	29	1	Mm2
......................................................................ugugaucUaacggauaggcug.................	3	1	Mm2
```

Star Mature

```
gaggccccuaucaugccucggccuguuuucuuucagcccaauccauguggaccauaugucagaaaaucaugugauccaacggauaggcugaaagcaaaacaaaaugc
.....................................................................ugugauccaacggauaggAug.................   2        1    Mm2
.....................................................................ugugauccaacgCauaggcug.................   1        1    Mm2
.....................................................................ugugaAccaacggauaggcug.................   1        1    Mm2
.....................................................................ugugauccaacggauaggcuU.................   298      1    Mm2
.....................................................................ugugauccaacggGuaggcug.................   1        1    Mm2
.....................................................................ugugauccaacggaGaggcug.................   1        1    Mm2
.....................................................................ugugauccUacggauaggcug.................   3        1    Mm2
.....................................................................uUugauccaacggauaggcug.................   4        1    Mm2
.....................................................................ugugauccaaUggauaggcug.................   5        1    Mm2
.....................................................................ugugauccaacggauGggcug.................   165      1    Mm2
.....................................................................ugugauccaacggUuaggcug.................   1        1    Mm2
.....................................................................ugugauccaacggaAaggcug.................   1        1    Mm2
.....................................................................ugugauccaacggaCaggcug.................   1        1    Mm2
.....................................................................ugugGuccaacggauaggcug.................   2        1    Mm2
.....................................................................ugugauccaacggauagAcug.................   47       1    Mm2
.....................................................................ugugauccaacggauaAgcug.................   16       1    Mm2
.....................................................................Ngugauccaacggauaggcug.................   6        1    Mm2
.....................................................................ugugauccaUcggauaggcug.................   1        1    Mm2
.....................................................................ugAgauccaacggauaggcug.................   3        1    Mm2
.....................................................................ugugCuccaacggauaggcug.................   1        1    Mm2
.....................................................................ugugauccaacggauaggcCg.................   4        1    Mm2
.....................................................................ugugauccaacggauaggcuC.................   2        1    Mm2
.....................................................................ugugauccaacggauaCgcug.................   16       1    Mm2
.....................................................................ugugauGcaacggauaggcug.................   1        1    Mm2
.....................................................................ugugauccaacgUauaggcug.................   1        1    Mm2
.....................................................................ugugauccaacggauaggcug.................   6493     0    Mm2
.....................................................................ugugauccaacggauaUgcug.................   11       1    Mm2
.....................................................................uCugauccaacggauaggcug.................   2        1    Mm2
.....................................................................ugugauccCacggauaggcug.................   2        1    Mm2
.....................................................................Agugauccaacggauaggcug.................   5        1    Mm2
.....................................................................ugugauccaacggauaggcuA.................   296      1    Mm2
.....................................................................ugugauUcaacggauaggcug.................   1        1    Mm2
.....................................................................ugugauccaacggauUggcug.................   1        1    Mm2
.....................................................................ugugaucGaacggauaggcug.................   1        1    Mm2
.....................................................................ugCgauccaacggauaggcug.................   1        1    Mm2
.....................................................................ugugauccaacUgauaggcug.................   1        1    Mm2
.....................................................................ugugauccaacAgauaggcug.................   2        1    Mm2
.....................................................................ugugauccGacggauaggcug.................   1        1    Mm2
.....................................................................uAugauccaacggauaggcug.................   6        1    Mm2
.....................................................................ugugauccaacggauaggcGg.................   187      1    Mm2
.....................................................................ugugauccaaGggauaggcug.................   14       1    Mm2
.....................................................................Cgugauccaacggauaggcug.................   3        1    Mm2
.....................................................................ugugauccaaAggauaggcug.................   9        1    Mm2
.....................................................................ugugauccaacCgauaggcuga................   7        1    Mm2
.....................................................................ugugauccaacggauagUcuga................   823      1    Mm2
.....................................................................ugugauccaacgAauaggcuga................   6        1    Mm2
.....................................................................ugugaucGaacggauaggcuga................   2        1    Mm2
.....................................................................ugugaucAaacggauaggcuga................   18       1    Mm2
.....................................................................ugugaNccaacggauaggcuga................   17       1    Mm2
.....................................................................ugugauccaacggauaggcuga................   163086   0    Mm2
.....................................................................ugugauccaacggauaggAuga................   12       1    Mm2
.....................................................................ugugauccaacggauaggcCga................   13       1    Mm2
.....................................................................uAugauccaacggauaggcuga................   71       1    Mm2
.....................................................................ugAgauccaacggauaggcuga................   40       1    Mm2
.....................................................................ugugauccaacggauaggcugU................   48479    1    Mm2
.....................................................................Agugauccaacggauaggcuga................   22       1    Mm2
.....................................................................ugCgauccaacggauaggcuga................   30       1    Mm2
.....................................................................ugugauccaaAggauaggcuga................   287      1    Mm2
.....................................................................ugugCuccaacggauaggcuga................   22       1    Mm2
.....................................................................uguUauccaacggauaggcuga................   12       1    Mm2
.....................................................................ugugauccaaGggauaggcuga................   171      1    Mm2
.....................................................................ugugauccaacggauagCcuga................   757      1    Mm2
.....................................................................ugugauccaacggauUggcuga................   16       1    Mm2
.....................................................................ugugaCccaacggauaggcuga................   15       1    Mm2
.....................................................................ugugauccaacggGuaggcuga................   15       1    Mm2
.....................................................................ugugauccaacggCuaggcuga................   9        1    Mm2
.....................................................................uUugauccaacggauaggcuga................   25       1    Mm2
.....................................................................ugugauccaaUggauaggcuga................   181      1    Mm2
.....................................................................ugugauccaGcggauaggcuga................   18       1    Mm2
.....................................................................ugugauccaacggauaggGuga................   6        1    Mm2
```

Star Mature

gaggccccuaucaugccucggccuguuuuccuuucagcccaauccauguggaccauaugucagaaaaucaugugauccaacggauaggcugaaagcaaaacaaaaugc			
....ugugauccaacggauaggcugC................	137	1	Mm2
....ugugauccaacAgauaggcuga................	36	1	Mm2
....ugugauccaacggUuaggcuga................	12	1	Mm2
....Ggugauccaacggauaggcuga................	5	1	Mm2
....ugugGuccaacggauaggcuga................	45	1	Mm2
....ugugaGccaacggauaggcuga................	7	1	Mm2
....uguAauccaacggauaggcuga................	28	1	Mm2
....ugugauccUacggauaggcuga................	77	1	Mm2
....ugugaAccaacggauaggcuga................	18	1	Mm2
....ugugauUcaacggauaggcuga................	26	1	Mm2
....ugugaucUaacggauaggcuga................	78	1	Mm2
....ugugauccaacggauaCgcuga................	428	1	Mm2
....ugugauccaUcggauaggcuga................	25	1	Mm2
....ugugauccaacggauaggUuga................	21	1	Mm2
....ugugauccaCcggauaggcuga................	15	1	Mm2
....ugugauccaacggauaAgcuga................	309	1	Mm2
....ugugauccaacggauagAcuga................	930	1	Mm2
....ugugauccaacggauaggcuUa................	26	1	Mm2
....ugugauccaacggauaggcuCa................	9	1	Mm2
....ugugauccaacggauCggcuga................	11	1	Mm2
....ugugauccCacggauaggcuga................	15	1	Mm2
....ugugauccaacggauaggcAga................	6	1	Mm2
....ugugauccaacggauaUgcuga................	262	1	Mm2
....uNugauccaacggauaggcuga................	5	1	Mm2
....ugugauccaacggaGaggcuga................	8	1	Mm2
....ugugauccaacgUauaggcuga................	27	1	Mm2
....ugugauccaacgCauaggcuga................	4	1	Mm2
....ugugauccaacggauaggcugG................	3288	1	Mm2
....ugugauccaacggauaggcuAa................	67	1	Mm2
....ugugauNcaacggauaggcuga................	12	1	Mm2
....uguCauccaacggauaggcuga................	8	1	Mm2
....ugugauccaacggaAaggcuga................	8	1	Mm2
....ugugUuccaacggauaggcuga................	22	1	Mm2
....ugugauccaacggauaggcGga................	4	1	Mm2
....ugGgauccaacggauaggcuga................	39	1	Mm2
....uCugauccaacggauaggcuga................	16	1	Mm2
....Cgugauccaacggauaggcuga................	19	1	Mm2
....ugugauGcaacggauaggcuga................	2	1	Mm2
....ugugauccaacggauGggcuga................	3220	1	Mm2
....ugugauccGacggauaggcuga................	45	1	Mm2
....ugugauAcaacggauaggcuga................	16	1	Mm2
....ugugauccaacUgauaggcuga................	27	1	Mm2
....Ngugauccaacggauaggcuga................	80	1	Mm2
....ugugauccaacggaCaggcuga................	17	1	Mm2
....ugugaucUaacggauaggcugaa...............	2	1	Mm2
....ugugauccUacggauaggcugaa...............	3	1	Mm2
....ugugGuccaacggauaggcugaa...............	1	1	Mm2
....ugugauccGacggauaggcugaa...............	1	1	Mm2
....ugugauccaacggauaggcAgaa...............	1	1	Mm2
....ugugauccaacggauagCcugaa...............	24	1	Mm2
....ugugauccaacggauaggcuAaa...............	8	1	Mm2
....ugugauccaacggUuaggcugaa...............	1	1	Mm2
....ugugauccaacggauagUcugaa...............	22	1	Mm2
....ugugauccaCcggauaggcugaa...............	2	1	Mm2
....uguUauccaacggauaggcugaa...............	2	1	Mm2
....uguAauccaacggauaggcugaa...............	1	1	Mm2
....ugugauccaacggauaggcugaG...............	40	1	Mm2
....ugugauccaaUggauaggcugaa...............	4	1	Mm2
....ugugauccaacggCuaggcugaa...............	2	1	Mm2
....ugugauccaacggauGggcugaa...............	111	1	Mm2
....Cgugauccaacggauaggcugaa...............	2	1	Mm2
....ugugauccaacggauaggcugaU...............	3311	1	Mm2
....ugugauccaacggauaggcugGa...............	1	1	Mm2
....ugugaCccaacggauaggcugaa...............	1	1	Mm2
....ugugauccaacggauaggcuUaa...............	1	1	Mm2
....ugugauccaacggauaCgcugaa...............	28	1	Mm2
....uUugauccaacggauaggcugaa...............	1	1	Mm2
....ugCgauccaacggauaggcugaa...............	2	1	Mm2
....ugugauccaacggauaggcCgaa...............	1	1	Mm2
....ugAgauccaacggauaggcugaa...............	3	1	Mm2

```
                                   Star                                 Mature
gaggccccuaucaugccucggccuguuuuccuuucagcccaauccauguggaccauaugucagaaaaucaugugauccaacggauaggcugaaagcaaaacaaaaugc
......................................................................ugugauccaacggaCaggcugaa...............   1     1   Mm2
......................................................................ugugauccaacggauaggcugUa...............   229   1   Mm2
......................................................................Ngugauccaacggauaggcugaa...............   1     1   Mm2
......................................................................ugugaNccaacggauaggcugaa...............   1     1   Mm2
......................................................................ugugauccaacAgauaggcugaa...............   1     1   Mm2
......................................................................ugugauccaGcggauaggcugaa...............   2     1   Mm2
......................................................................ugugauccaacggauUggcugaa...............   1     1   Mm2
......................................................................ugugauccaaGggauaggcugaa...............   9     1   Mm2
......................................................................ugugauccaacggauaUgcugaa...............   19    1   Mm2
......................................................................uCugauccaacggauaggcugaa...............   1     1   Mm2
......................................................................ugugauccaacggauaAgcugaa...............   8     1   Mm2
......................................................................ugugauccaacggauaggcugaa...............   5918  0   Mm2
......................................................................ugGgauccaacggauaggcugaa...............   3     1   Mm2
......................................................................ugugauccaaAggauaggcugaa...............   10    1   Mm2
......................................................................ugugauccaUcggauaggcugaa...............   1     1   Mm2
......................................................................uguCauccaacggauaggcugaa...............   1     1   Mm2
......................................................................Agugauccaacggauaggcugaa...............   3     1   Mm2
......................................................................ugugauccaacggauagAcugaa...............   42    1   Mm2
......................................................................ugugauccaacggauaggcugaC...............   10    1   Mm2
......................................................................ugugauccaaGggauaggcugaaa..............   2     1   Mm2
......................................................................ugugauccaacggauagCcugaaa..............   5     1   Mm2
......................................................................ugugauccaacggauGggcugaaa..............   15    1   Mm2
......................................................................ugGgauccaacggauaggcugaaa..............   1     1   Mm2
......................................................................ugugauccaacggauaCgcugaaa..............   2     1   Mm2
......................................................................ugugauccGacggauaggcugaaa..............   1     1   Mm2
......................................................................ugugauccaaUggauaggcugaaa..............   1     1   Mm2
......................................................................ugugauccaacggauaggcugaUa..............   82    1   Mm2
......................................................................ugugauccaacggauagUcugaaa..............   1     1   Mm2
......................................................................Agugauccaacggauaggcugaaa..............   1     1   Mm2
......................................................................ugugaucGaacggauaggcugaaa..............   1     1   Mm2
......................................................................ugugauccaacggauaggcugaaC..............   15    1   Mm2
......................................................................ugugauccaacggauaggcugUaa..............   21    1   Mm2
......................................................................ugugauccaacggauaggcugaaU..............   469   1   Mm2
......................................................................ugugauccaacggauaggcugaaa..............   680   0   Mm2
......................................................................uguCauccaacggauaggcugaaa..............   1     1   Mm2
......................................................................ugugauccaacgUauaggcugaaa..............   1     1   Mm2
......................................................................ugugauccaacggauaggcugaGa..............   24    1   Mm2
......................................................................ugugauccaacggauagAcugaaa..............   4     1   Mm2
......................................................................ugugauccaacggauaggcuUaaa..............   1     1   Mm2
......................................................................ugugauGcaacggauaggcugaaa..............   1     1   Mm2
......................................................................ugugauccaacggauaggcugaaG..............   61    1   Mm2
......................................................................ugugauUcaacggauaggcugaaa..............   1     1   Mm2
......................................................................ugugauccaacggauaggcugaGag.............   1     1   Mm2
......................................................................ugugauccaacggauaggcugaaGg.............   1     1   Mm2
......................................................................ugugauccaacggauaggcugaaaA.............   47    1   Mm2
......................................................................ugugauccaacggauaggcugaaag.............   11    0   Mm2
......................................................................ugugauccaacggauaggcugaaaC.............   2     1   Mm2
......................................................................ugugauccaacggauaggcugaaaU.............   95    1   Mm2
......................................................................ugugauccaacggauaggcugaaagU............   21    1   Mm2
......................................................................ugugauccaacggauaggcugaaagA............   1     1   Mm2
......................................................................ugugauccaacggauaggcugaaagGa...........   1     1   Mm2
......................................................................ugugauccaacggauaggcugaaagAa...........   1     1   Mm2
.......................................................................gugauccaacggauaggc...................   1     0   Mm2
.......................................................................gugauccaacggauaggcu..................   1     0   Mm2
.......................................................................gugauccaacggauaggcug.................   7     0   Mm2
.......................................................................gugauccaacggauaggcugU................   14    1   Mm2
.......................................................................gugauccaacggauaggcugG................   1     1   Mm2
.......................................................................gugauccaacggauaggUuga................   1     1   Mm2
.......................................................................gugauccaacggauaggcuga................   45    0   Mm2
.......................................................................Uugauccaacggauaggcuga................   1     1   Mm2
.......................................................................gugauccaCcggauaggcuga................   1     1   Mm2
.......................................................................gugauccaacggauaggcugaU...............   1     1   Mm2
........................................................................ugauccaacggauaggcug.................   1     0   Mm2
........................................................................ugauccaacggauGggcuga................   1     1   Mm2
........................................................................ugauccaacggauaggcugU................   8     1   Mm2
........................................................................ugauccaacggauaggcuga................   7     0   Mm2
.........................................................................gauccaacggauaggcug.................   2     0   Mm2
.........................................................................Uauccaacggauaggcuga................   1     1   Mm2
.........................................................................gauccaacggauaggcugU................   1     1   Mm2
..........................................................................auccaacggauaggcugaaagc............   1     0   Mm2
```

```
                                   Star                     Mature
gaggccccuaucaugccucggccuguuuuccuuucagcccaauccauguggaccauaugucagaaaaucaugugauccaacggauaggcugaaagcaaaacaaaaugc

...................................agcccaauccauguggac.......................................................   11     0     Mm1
...................................agcccaauccauguggaccaua...................................................   7      0     Mm1
...................................agcccaauccauguggaccauau..................................................   8      0     Mm1
...................................agcccaauccauguggCccauau..................................................   1      1     Mm1
....................................gcccaauccauguggaccauaug.................................................   2      0     Mm1
.....................................................................Cugugauccaacggauaggcugaa...............   9      1     Mm1
......................................................................ugugauccaaUggauagg....................   1      1     Mm1
......................................................................ugugauccaUcggauagg....................   1      1     Mm1
......................................................................ugugauccaacggauagA....................   49     1     Mm1
......................................................................ugugauccaacggauagU....................   118    1     Mm1
......................................................................ugugauccaacggauagg....................   154    0     Mm1
......................................................................ugugauccaacggauaggU...................   28     1     Mm1
......................................................................ugugauccaacggauaUgc...................   1      1     Mm1
......................................................................ugugauccUacggauaggc...................   1      1     Mm1
......................................................................ugugauccaacggauagCc...................   1      1     Mm1
......................................................................ugugauccaacggauaggc...................   335    0     Mm1
......................................................................ugugauccaaGggauaggc...................   1      1     Mm1
......................................................................ugugauccaacggauaAgc...................   1      1     Mm1
......................................................................ugugauccaacggauagUc...................   3      1     Mm1
......................................................................ugugauccaacggauaCgc...................   1      1     Mm1
......................................................................ugugauccaacggauagAc...................   3      1     Mm1
......................................................................ugugauccaaAggauaggcu..................   4      1     Mm1
......................................................................ugugauNcaacggauaggcu..................   1      1     Mm1
......................................................................Ngugauccaacggauaggcu..................   3      1     Mm1
......................................................................ugugauccUacggauaggcu..................   2      1     Mm1
......................................................................ugugaCccaacggauaggcu..................   2      1     Mm1
......................................................................ugugauccaacggauagUcu..................   20     1     Mm1
......................................................................ugugauccaGcggauaggcu..................   2      1     Mm1
......................................................................ugugauccaacggauGggcu..................   102    1     Mm1
......................................................................ugCgauccaacggauaggcu..................   1      1     Mm1
......................................................................ugugauccaacggauaCgcu..................   16     1     Mm1
......................................................................ugugauccaacggauaggcC..................   1      1     Mm1
......................................................................Ggugauccaacggauaggcu..................   1      1     Mm1
......................................................................ugugauccaacggaAaggcu..................   1      1     Mm1
......................................................................ugGgauccaacggauaggcu..................   1      1     Mm1
......................................................................ugugauccaacggUuaggcu..................   1      1     Mm1
......................................................................ugugauccaacggauaggcG..................   24     1     Mm1
......................................................................ugugauccaacggaGaggcu..................   1      1     Mm1
......................................................................ugugauccaacggauagCcu..................   20     1     Mm1
......................................................................ugugaNccaacggauaggcu..................   1      1     Mm1
......................................................................ugugauccaacggauaUgcu..................   5      1     Mm1
......................................................................ugugauccaacggauagAcu..................   21     1     Mm1
......................................................................ugugauccaacggauaggcA..................   14     1     Mm1
......................................................................ugugauccaaGggauaggcu..................   3      1     Mm1
......................................................................ugugauccGacggauaggcu..................   1      1     Mm1
......................................................................ugugauccaacggauaAgcu..................   10     1     Mm1
......................................................................uAugauccaacggauaggcu..................   2      1     Mm1
......................................................................ugugauccaaUggauaggcu..................   4      1     Mm1
......................................................................Agugauccaacggauaggcu..................   1      1     Mm1
......................................................................ugugauccaacggauaggcu..................   4162   0     Mm1
......................................................................ugugauccaacggauagAcug.................   17     1     Mm1
......................................................................ugugauccaacggGuaggcug.................   1      1     Mm1
......................................................................Agugauccaacggauaggcug.................   1      1     Mm1
......................................................................ugugauccaaAggauaggcug.................   1      1     Mm1
......................................................................ugugauccaaUggauaggcug.................   2      1     Mm1
......................................................................ugugaucUaacggauaggcug.................   1      1     Mm1
......................................................................ugugauccaacgCauaggcug.................   1      1     Mm1
......................................................................ugugauccUacggauaggcug.................   4      1     Mm1
......................................................................ugugauccaacUgauaggcug.................   1      1     Mm1
......................................................................ugugauccaacggauaggcuA.................   37     1     Mm1
......................................................................Cgugauccaacggauaggcug.................   2      1     Mm1
......................................................................ugugauccaacggauaggcug.................   2543   0     Mm1
......................................................................ugugauccaacggauaCgcug.................   6      1     Mm1
......................................................................ugugauccaacCgauaggcug.................   1      1     Mm1
......................................................................ugugauccCacggauaggcug.................   1      1     Mm1
......................................................................ugugauUcaacggauaggcug.................   1      1     Mm1
......................................................................ugGgauccaacggauaggcug.................   2      1     Mm1
......................................................................ugugauccaacggauaggcuU.................   39     1     Mm1
......................................................................ugugauccaacggauGggcug.................   80     1     Mm1
```

Star   Mature

```
gaggccccuaucaugccucggccuguuuuccuuucagcccaauccauguggaccauaugucagaaaaucaugugauccaacggauaggcugaaagcaaaacaaaaugc
......................................................................ugugauccaacggauaUgcug.................    6      1    Mm1
......................................................................ugugauccaaGggauaggcug.................    4      1    Mm1
......................................................................ugugauccaacggauaggcAg.................    1      1    Mm1
......................................................................ugugauccaacggauaggcuC.................    11     1    Mm1
......................................................................ugugauccaacggauagUcug.................    12     1    Mm1
......................................................................ugugauccaacggauagCcug.................    12     1    Mm1
......................................................................ugugauGcaacggauaggcug.................    1      1    Mm1
......................................................................ugugauccaacggauaAgcug.................    4      1    Mm1
......................................................................ugugauccaacggauaggcGg.................    4      1    Mm1
......................................................................ugugauccaacgAauaggcug.................    1      1    Mm1
......................................................................Ngugauccaacggauaggcug.................    1      1    Mm1
......................................................................ugAgauccaacggauaggcuga................    10     1    Mm1
......................................................................ugugauccaacggaGaggcuga................    1      1    Mm1
......................................................................ugugaucGaacggauaggcuga................    1      1    Mm1
......................................................................Agugauccaacggauaggcuga................    3      1    Mm1
......................................................................ugugaGccaacggauaggcuga................    1      1    Mm1
......................................................................ugugauccaacggauaggcuUa................    6      1    Mm1
......................................................................ugugauGcaacggauaggcuga................    2      1    Mm1
......................................................................ugugaNccaacggauaggcuga................    4      1    Mm1
......................................................................Cgugauccaacggauaggcuga................    5      1    Mm1
......................................................................ugugauccaacCgauaggcuga................    1      1    Mm1
......................................................................ugugauccaacgUauaggcuga................    5      1    Mm1
......................................................................ugugauccaacAgauaggcuga................    9      1    Mm1
......................................................................Ngugauccaacggauaggcuga................    20     1    Mm1
......................................................................ugugauccaGcggauaggcuga................    7      1    Mm1
......................................................................ugugauccaaGggauaggcuga................    51     1    Mm1
......................................................................ugGgauccaacggauaggcuga................    9      1    Mm1
......................................................................ugugaAccaacggauaggcuga................    4      1    Mm1
......................................................................ugugauccGacggauaggcuga................    5      1    Mm1
......................................................................ugugauccaacggauaggUuga................    8      1    Mm1
......................................................................ugugauccaacggauaCgcuga................    93     1    Mm1
......................................................................ugugUuccaacggauaggcuga................    3      1    Mm1
......................................................................uguCauccaacggauaggcuga................    1      1    Mm1
......................................................................ugugauccaacggauagUcuga................    179    1    Mm1
......................................................................ugugauccaacgAauaggcuga................    1      1    Mm1
......................................................................ugugauccaacggaCaggcuga................    3      1    Mm1
......................................................................ugugauccaacggauaggGuga................    1      1    Mm1
......................................................................ugugaCccaacggauaggcuga................    4      1    Mm1
......................................................................ugugauccaacggauaggcAga................    3      1    Mm1
......................................................................uUugauccaacggauaggcuga................    5      1    Mm1
......................................................................ugugaucAaacggauaggcuga................    2      1    Mm1
......................................................................ugugauccaacggauagAcuga................    209    1    Mm1
......................................................................ugugauAcaacggauaggcuga................    3      1    Mm1
......................................................................ugugauNcaacggauaggcuga................    4      1    Mm1
......................................................................ugugauccaacggUuaggcuga................    2      1    Mm1
......................................................................ugugCuccaacggauaggcuga................    9      1    Mm1
......................................................................ugCgauccaacggauaggcuga................    3      1    Mm1
......................................................................ugugauccaCcggauaggcuga................    4      1    Mm1
......................................................................ugugauccaacggauaggAuga................    3      1    Mm1
......................................................................ugugauccaUcggauaggcuga................    3      1    Mm1
......................................................................ugugauccaacggauUggcuga................    4      1    Mm1
......................................................................uguUauccaacggauaggcuga................    3      1    Mm1
......................................................................ugugauccaacggCuaggcuga................    1      1    Mm1
......................................................................ugugauccaacUgauaggcuga................    7      1    Mm1
......................................................................ugugauccaacggauagCcuga................    191    1    Mm1
......................................................................ugugauccaacggauaggcuga................    37211  0    Mm1
......................................................................ugugauccaacggauaAgcuga................    94     1    Mm1
......................................................................ugugauccUacggauaggcuga................    20     1    Mm1
......................................................................ugugauccaacgCauaggcuga................    2      1    Mm1
......................................................................ugugauccaacggauaggcGga................    1      1    Mm1
......................................................................ugugauUcaacggauaggcuga................    6      1    Mm1
......................................................................ugugauccaacggauaggcuCa................    3      1    Mm1
......................................................................ugugauccCacggauaggcuga................    3      1    Mm1
......................................................................ugugauccaaUggauaggcuga................    44     1    Mm1
......................................................................ugugauccaacggauaggcugC................    25     1    Mm1
......................................................................ugugauccaacggauaggcugU................    7383   1    Mm1
......................................................................ugugauccaacggauaUgcuga................    64     1    Mm1
......................................................................ugugauccaaAggauaggcuga................    65     1    Mm1
......................................................................ugugGuccaacggauaggcuga................    14     1    Mm1
......................................................................uguAauccaacggauaggcuga................    7      1    Mm1
```

```
                                   Star                                  Mature
gaggccccuaucaugccucggccuguuuuccuuucagcccaauccauguggaccauaugucagaaaaucaugugauccaacggauaggcugaaagcaaaacaaaaugc
......................................................................uCugauccaacggauaggcuga................ 3      1    Mm1
......................................................................ugugauccaacggauaggcugG................ 156    1    Mm1
......................................................................uAugauccaacggauaggcuga................ 14     1    Mm1
......................................................................ugugaucUaacggauaggcuga................ 16     1    Mm1
......................................................................ugugauccaacggauaggcCga................ 8      1    Mm1
......................................................................ugugauccaacggauaggcuAa................ 4      1    Mm1
......................................................................ugugauccaacggGuaggcuga................ 1      1    Mm1
......................................................................ugugauccaacggauGggcuga................ 1009   1    Mm1
......................................................................ugugauccaacggauaggcugaG............... 25     1    Mm1
......................................................................ugugauccaacggauGggcugaa............... 25     1    Mm1
......................................................................ugugauccaacggauaCgcugaa............... 12     1    Mm1
......................................................................uCugauccaacggauaggcugaa............... 1      1    Mm1
......................................................................ugugauccaacggauagCcugaa............... 11     1    Mm1
......................................................................ugugCuccaacggauaggcugaa............... 1      1    Mm1
......................................................................ugGgauccaacggauaggcugaa............... 1      1    Mm1
......................................................................uguAauccaacggauaggcugaa............... 2      1    Mm1
......................................................................ugugauccaacggauaggcugaa............... 2858   0    Mm1
......................................................................ugugauccaaUggauaggcugaa............... 2      1    Mm1
......................................................................ugugauccaacggauaggcAgaa............... 1      1    Mm1
......................................................................ugugauccaaGggauaggcugaa............... 8      1    Mm1
......................................................................ugugauccaacggauagUcugaa............... 15     1    Mm1
......................................................................ugugaucGaacggauaggcugaa............... 1      1    Mm1
......................................................................ugugauccaacggauaUgcugaa............... 8      1    Mm1
......................................................................ugugauccaacggauaggcCgaa............... 1      1    Mm1
......................................................................uAugauccaacggauaggcugaa............... 1      1    Mm1
......................................................................ugugauccaacggauaggcugUa............... 54     1    Mm1
......................................................................Agugauccaacggauaggcugaa............... 2      1    Mm1
......................................................................ugugauccaacggauaAgcugaa............... 8      1    Mm1
......................................................................uUugauccaacggauaggcugaa............... 1      1    Mm1
......................................................................ugugaucUaacggauaggcugaa............... 2      1    Mm1
......................................................................ugugauccaacggauaggcugaU............... 625    1    Mm1
......................................................................Ggugauccaacggauaggcugaa............... 1      1    Mm1
......................................................................ugugauccaacggauagAcugaa............... 18     1    Mm1
......................................................................ugugauccaUcggauaggcugaa............... 2      1    Mm1
......................................................................ugugauccaacggauaggcugaaU.............. 166    1    Mm1
......................................................................ugugauccaacggauagAcugaaa.............. 1      1    Mm1
......................................................................ugugauccaaUggauaggcugaaa.............. 1      1    Mm1
......................................................................uAugauccaacggauaggcugaaa.............. 1      1    Mm1
......................................................................ugugauccaacggauaggcugaGa.............. 1      1    Mm1
......................................................................ugugauccaacggauaggcugaaC.............. 1      1    Mm1
......................................................................ugugauccaacggauaggcugaaa.............. 173    0    Mm1
......................................................................ugugauccaacggauaCgcugaaa.............. 2      1    Mm1
......................................................................ugugauccaacggauaggcugaaG.............. 6      1    Mm1
......................................................................ugugauccaacggauaggcugaUa.............. 12     1    Mm1
.......................................................................gugauccaacggauaggcugU................ 3      1    Mm1
.........................................................................gauccaacggauaggcug................. 9      0    Mm1

......................................................................ugugauccaacggauaggU................... 1      1    Fm1
......................................................................ugugauccaacggauaggcu.................. 7      0    Fm1
......................................................................ugugauccaacggauGggcu.................. 1      1    Fm1
......................................................................ugugauccaacggauaggcug................. 10     0    Fm1
......................................................................ugugauccaacggauaAgcug................. 1      1    Fm1
......................................................................ugugauccaacCgauaggcug................. 1      1    Fm1
......................................................................ugugauccaacggauagUcuga................ 1      1    Fm1
......................................................................ugugauccaacggauaggcugC................ 8      1    Fm1
......................................................................ugugauccaaAggauaggcuga................ 1      1    Fm1
......................................................................ugugauccaacggauaggcugU................ 26     1    Fm1
......................................................................ugugauccaacggauGggcuga................ 14     1    Fm1
......................................................................ugugauccaacggauagAcuga................ 1      1    Fm1
......................................................................ugAgauccaacggauaggcuga................ 1      1    Fm1
......................................................................ugugauccaacggauaggcuga................ 108    0    Fm1
......................................................................ugugauccaacggauaggcugaU............... 2      1    Fm1
......................................................................ugugauccaacggauaggcugaa............... 3      0    Fm1
......................................................................ugugauccaacggauaggcugUa............... 1      1    Fm1
......................................................................ugugauccaacggauaggcugaaU.............. 2      1    Fm1

......................................................................ugugauccaacggauaggc................... 2      0    Fm2
......................................................................ugugauccaacggauaggcu.................. 9      0    Fm2
......................................................................ugugauccaacggauaggcug................. 6      0    Fm2
......................................................................ugugauccaacggauaggcugU................ 34     1    Fm2
```

Star **Mature**

```
gaggccccuaucaugccucggccuguuuuccuuucagcccaauccauguggaccauaugucagaaaaucaugugauccaacggauaggcugaaagcaaaacaaaaugc
......................................................................ugugauccaacggauGggcuga................   32   1   Fm2
......................................................................ugugauccaacggauagCcuga................   1    1   Fm2
......................................................................ugugauccaacggauaggcuga................   72   0   Fm2
......................................................................ugugauccaacggauaggcugG................   2    1   Fm2
......................................................................Agugauccaacggauaggcuga................   1    1   Fm2
......................................................................ugugauccaacggauaggcugaU...............   4    1   Fm2
......................................................................ugugauccaacggauaggcugaa...............   9    0   Fm2
```
